# Notes on the Anatomy, Physiology and Pathological Significance of Pain and Other Nervous Phenomena in Relation to Dental Disease

**Published:** 1891-09

**Authors:** Thomas Gaddes


ARTICLE V.

### NOTES ON THE ANATOMY, PHYSIOLOGY AND PATHOLOGICAL SIGNIFICANCE OF PAIN AND OTHER NERVOUS PHENOMENA IN RELATION TO DENTAL DISEASE.

BY THOMAS GADDES, M. D., L. D. S., ENG. AND EDIN.

A paper read at the June meeting of the Colorado State Dental Association, and by the courtesy of the Editor of the *Dental Review* sent as an advanced proof to the *Dental Record.*

It may be said without fear of controversy that no one of the sciences upon which medicine is founded has advanced more within recent years than physiology has done. And perhaps the physiology of the nervous system, considering its supreme complexity and the difficulties attending its pursuit, stands out not the least prominent with its luminous strata unearthed by recent discovery. Of the greatest importance to the practice of medicine and surgery, is a knowledge of blood pressure in the systemic vessels as controlled through the influence of the vaso-motor upon the small arteries and veins. And the latest induction in this relation is the existence of a similar vaso-motor and controlling function presiding over the pulmonary system of blood vessels. Almost incalculable also is the value of the physiological data spoken of as cerebral localization—that is the knowledge of the definite situations upon the surface or gray matter of the cerebrum which are the nerve centres controlling, through the mind, certain muscles and groups of muscles, as those of speech, of facial expression, of the arm, the leg, &c. How those centres, like electric batteries, are connected with one another; that they have a certain resistence to discharge; that their stored up energy may be caused to overflow to other centres or cells; that those centres situate high up in the brain are connected with others at lower levels and less associated with psychical influence; that the higher centres can be inhibited, restrained or switched out of connection with one another and with lower centres, and so on. Consequent upon those increments to knowledge, many of the explanations of perverted or diseased action have to be remodelled, not upon the old lines simply, but, in many instances, upon a totally different foundation.

This information necessarily relates to the dentist as well as to the surgeon and the practitioner of medicine; each having the science of physiology for a partial, yet most important, basis of his sphere of action.

Let me preface what follows with these anatomical facts relating to nerve tissues :—

A nerve fibril between its peripheral and its central terminations is not branched, nor does it communicate.

The fibres forming several nerve trunks may be re-arranged as in a plexus, but the individual and ultimate fibrils of an axis cylinder do not branch. Any branching that may take place is probably limited to their peripheral and central endings.

The question may arise in the intelligence of some one as to how there can be an anatomy of pain as set forth in the title to this paper. It will be well, therefore, to clear up any such possible uncertainty.

Pain is not a simple, but a compound nervous phenomenon. Pain is a condition of the feelings, and therefore, of consciousness. It is subjective. Its cause may be objective—as an exposed pulp, the extraction of a tooth; or the cause may be subjective, as in hysteria, the hypnotic and the ideal states. It is affirmed, and truly so, that the phenomena of pleasure and pain are perhaps the most obscure and involved which psychology includes. Notwithstanding that the structural units out of which pleasure and pain are built, admit of being studied in their individualities and in their aggregations; and also in their simple relations and in their complex relations.

According to Fechner's psycho-physical law, sensations increase proportionately to the logarithms of the strength of the stimulus, and as we speak of the pulse wave as traced by the sphygmograph in terms of motion, we can also set forth the different aspects of feeling in terms of matter and motion. The following sketch diagramatically presents an incomplete analysis of feeling and the equivalent expressed in physical terms:

| | | | |
|---|---|---|---|
| Feeling varies | According to cause, which may be | | repeated—number. |
| | | | different—quantity. |
| | | | varied in degree—quality. |
| | According to course of impulse, which may be | peripheral | thro' one fibril. |
| | | | thro' several fibrils. |
| | | centripetal | from one centre. |
| | | | from higher planes of centres—diffused. |
| | In character, as in its | | intensity—amplitude. |
| | | | duration—time. |
| | | | frequency of recurrence—time. |

All nerve stimuli are motions, so too are all nerve impulses. Those rapidly recurring and exceedingly delicate nerve shocks; those pulses of molecular change which are made through the nerve substance of a sentient centre are regarded as the units of feeling or consciousness. A simple feeling or sensation is composed of units of feeling. A shock or pulse (which eventually produces a unit of feeling) being repeatedly caused, being differently caused, and having varying degrees of causation, and reaching the centre by different routes, and by acting upon other centres, thus, it is asserted, gives rise to combinations of simple feelings, and these again are further compounded, ascending to higher and yet higher strata of nerve cells with the result of there being produced feelings of extreme complexity and heterogeneity both in structure and in relation. G. H. Lewes asserts that sensibility increases according to the extent of the ganglia affected.

Let us ascertain what light can be thrown upon the question by analogy. A given flute note has certain physiological qualities known as pitch, intensity and timbre. A note of a clarionet may differ in each of those qualities. The two physiological sounds are represented by physical (ærial) vibrations of essentially different yet corresponding properties. The pitch has its wave length; the intensity its amplitude; the timbre its secondary harmonic components or overtones. Each physiological effect is represented by vibrations of definite form and structure. The musical sound is the result of the combination of these structures. There is thus an anatomy of sound. Or, again, in listening to a well-trained choir we hear the several parts in harmonic relations; each part having its own melody, *crescendo*, *diminuendo*, rests and other variations. Those components have certain effects upon the mind as they are perceived through the auditory apparatus. It is thus ordinarily. But the acutely organized musician can realize and produce more or less completely similar effects upon his mind, not by hearing the sounds, but by reading their symbols as presented in the musical score. The notation is the symbolic representation of the musical sounds, harmonies, etc. The chemist, too, represents the composition and structure of compounds by his symbols; and the isomeric changes, motions or functions of certain substances he also presents to his mind by his graphic formulæ. The astronomer, likewise by his mass of algebraical calculations, symbolizes the molar motions of the sun, moon and planets. In all these instances there is a symbolization expressing the motions of matter. As those symbols have structural relations, the study of those relations is the study of their anatomy.

Keeping in view the biological axiom that function and structure are correlatives, and that complexity of function implies complexity of structure, we can very readily realize that such a compound combination of feelings as is synonymous with the musical sound just instanced, or with ordinary pain, admits of being studied morphologically—admits of an anatomy. The nature of that anatomy has been indicated.

To resolve the question of the anatomy of pain and other nervous phenomena into its lowest terms I venture to advance the following proposition and induction :

All nerve stimuli and all nerve impulses are motions.

Motion implies a something moved.

Motion, occurring in space and time, is the function of the something—matter.

The phenomena of motion can be presented to the mind by physical symbols.

Physical symbols—as to form, as to quantity, as to space, as to time, in their several relations—therefore represent the physical aspects of the anatomy of nerve phenomena.

I would have it distinctly understood that in this paper I am not dealing with the nature or relation of the ego.

Pain may be studied in its physiological and in its psychological aspects. All nerve stimuli are motions, so, too, are all nerve impulses. When those motions can be interpreted by physical means they are, therefore, classed as physiological; but when those nerve disturbances result in or are followed by subjective phenomena that are psychically interpreted as feelings, then, it is claimed, the domain of psychology is reached. Just as we saw that the cause of pain may be objective and subjective, so also are the antecedent nerve disturbances of pain objective while the effect produced upon the centres (in consciousness) is subjective and known as a unit of feeling, a simple feeling, or a complex feeling—pleasure or pain. Analyzed in this way it becomes apparent that pain has its physiological or objective side, and its psychological or subjective side.

We are now in a position to state that pain caused by an exposed tooth pulp is not situate in that pulp, as is ordinarily expressed. The state of feeling known as pain has the nerve centre for its seat rather than the nerve periphery. We commonly say that we have pain in the finger when it receives a misdirected blow from a hammer, or in a toe when a tender corn meets with the clumsy tread of a well meaning friend. Strictly speaking, the disturbance commencing at the periphery, and becoming augmented in its progress, is received at, and perceived—makes its impression upon consciousness—by means of, the nerve centre. A particular mass of vesicular matter forming the central end of a nerve always receives the impulses from the periphery of that nerve; and there has become established a definite relation between the excitation of such a centre and the peripheral ending of the fibril connected with it. In order to make this quite clear let me restate the case. Ordinary impulses travelling along a given nerve fibre from its periphery to its centre always, in the normal condition, arrive at a certain cell; and by such action, repeated myriads of times and through countless ancestors, a definite cell area becomes the associated centre of a definite fibril. So that on just sufficiently exciting one such central area, or the fibril leading to it, there arise in consciousness those phenomena of sensation and of location similarly as when the periphery itself were the seat of stimulation. Familiar example of this latter is the pain in the third and fourth fingers on striking the ulnar nerve, or "funny bone," at the elbow; also the pain in the toes of an amputated leg. On the other hand, an irritation of a certain area of the brain—as by a tumor, or splinter of bone, or electricity—gives rise to similar sensory or motor disturbances. But let the excitation of the centre be too great, or the centre itself be surcharged as it were, and then the impulse overflows to other cells and excites them. Thus sensation or pain is referred to quite different regions from that which is the seat of stimulation. Witness for instance a disease confined to one tooth giving rise to pain in several teeth; congestion of the liver, pain between the shoulders; and the various nerve tracks along which neuralgia is often manifested. Or, instead of overflowing to contiguous sensory centres, or even perverting the function of the special centres, motor areas may be excited resulting in reflex muscular twitchings, clonic and tonic contractions, convulsions, and even epileptic seizures.

It will now be perfectly clear that sensation, or the reception into consciousness of sensory impulses, is the function of nerve cells. Therefore pain as a form of sensation is not situate in the periphery, as in a diseased canine tooth, but necessarily in the nerve centre. How can the pain which the patient "feels" in the toes of the amputated foot have its seat in those defunct and perhaps buried members?

In strict interpretation of the phenomenon, I do not feel pain in an inflamed pulp; but that sensation in consciousness which I interpret as pain I *refer* to the seat of inflammation, or *refer* to the lost toe as being the wonted termination of the divided nerve fibre in the stump; or *refer* to the ear in the not infrequent case of overflow from the cells of the inferior dental nerve to the neighboring central cells which supply the external auditory meatus, thus giving rise to earache.

Pain having an anatomical, a physiological and a psychological basis, serves as an index of a pathological state. The pathological state may be in the nerve peripheries, fibres, cells, or substance (neuralgia). It may be extra-neural, *i. e.*, primary affections of tissues beyond the nervous structures. It may be central, as in delusions, hysteria, and in the hypnotized. In these various ways pain is an indicator of something wrong.

From what has already been said it will be apparent that the individual who suffers pain is by no means a reliable judge of the seat of disease. Patients look upon the seat of pain as the necessary seat of disease. That this is so, recall the several examples already noticed as evidence; also the pain in the knee in hip joint disease; likewise eruptions of the face and scalp in the teething process of children; cases of half the tongue furred, ulceration of the external auditory meatus; affections of the eye, perverted taste—all these latter are often secondary affections arising from nervous disturbances caused by the condition of the teeth. But this view, that the supposed seat of pain is the region of disease, is more frequently wrong than right, and the practitioner should ever be on the alert, While he interprets the true significance of pain, he must avoid being led away by the patient's localization. Who would dream of treating a varicose ulcer without first giving attention to the condition of distant blood vessels? It is the charlatan who treats effects and ignores causes. Having ascertained the cause of the pain, of the ulcer, of the convulsion—which may be immediate, mediate or remote—and directed treatment for its removal, it is good practice, and practice based upon anatomical and physiological grounds, to apply remedies to the area where morbid action is manifest. For example: A patient complains of pain in the ear; the cause is diagnosed as a faulty lower molar. While the tooth is being treated, which may take some time ere relief is found, benefit not infrequently quickly follows the application of an anodyne to the external auditory meatus. The nerve fibril from the tooth has its central cell or vesicle connected or associated with the central cell of the auriculo-temporal nerve supplying the ear. In virtue of this connection, pain is transmitted to that cell and referred to the ear. It is found in practice that by applying remedies to the area of distribution of associated nerves relief of pain often follows. This is so with regard to the case of the ear, as just cited; likewise with the application of thymol, veratria, aconite, etc., to the course and distribution of a nerve in neuralgia. Also in the treatment of inflammation by counter irritation—the counter irritant inflames the surface to which it is applied, and through the vaso-motor nerves the distant vessels at the seat of critical inflammation are influenced.

It is the possession of what knowledge is attainable as to the *modus operandi* of methods of the kind here noted that distinguishes the educated practitioner from the mere charlatan. Knowledge and the scientific method of thought and investigation are the royal roads to correct diagnosis, successful treatment and professional honor.—*London Dental Record.*

